# Dynamic programming re-ranking for PPI interactor and pair extraction in full-text articles

**DOI:** 10.1186/1471-2105-12-60

**Published:** 2011-02-23

**Authors:** Richard Tzong-Han Tsai, Po-Ting Lai

**Affiliations:** 1 Department of Computer Science & Engineering, Yuan Ze University, Chung-Li, Taiwan, R.O.C

## Abstract

**Background:**

Experimentally verified protein-protein interactions (PPIs) cannot be easily retrieved by researchers unless they are stored in PPI databases. The curation of such databases can be facilitated by employing text-mining systems to identify genes which play the interactor role in PPIs and to map these genes to unique database identifiers (interactor normalization task or INT) and then to return a list of interaction pairs for each article (interaction pair task or IPT). These two tasks are evaluated in terms of the area under curve of the interpolated precision/recall (AUC iP/R) score because the order of identifiers in the output list is important for ease of curation.

**Results:**

Our INT system developed for the BioCreAtIvE II.5 INT challenge achieved a promising AUC iP/R of 43.5% by using a support vector machine (SVM)-based ranking procedure. Using our new re-ranking algorithm, we have been able to improve system performance (AUC iP/R) by 1.84%. Our experimental results also show that with the re-ranked INT results, our unsupervised IPT system can achieve a competitive AUC iP/R of 23.86%, which outperforms the best BC II.5 INT system by 1.64%. Compared to using only SVM ranked INT results, using re-ranked INT results boosts AUC iP/R by 7.84%. Statistical significance t-test results show that our INT/IPT system with re-ranking outperforms that without re-ranking by a statistically significant difference.

**Conclusions:**

In this paper, we present a new re-ranking algorithm that considers co-occurrence among identifiers in an article to improve INT and IPT ranking results. Combining the re-ranked INT results with an unsupervised approach to find associations among interactors, the proposed method can boost the IPT performance. We also implement score computation using dynamic programming, which is faster and more efficient than traditional approaches.

## Background

Biological databases, such as pathway databases, are very useful in helping biologists discover new and existing biological mechanisms. Each entry in a database is based on experimental results. Yet as the amount of published research increases, constructing databases becomes more difficult. Biomedical text mining is a key technology for automatically extracting important information from biomedical literature.

One key task in biomedical text mining is gene normalization (GN), mapping genes mentioned in the text to their unique database IDs. This task is difficult because one gene mention may map to different genes in various species. For example, papers containing *in vivo *experiments may describe mouse and human genes with the same name (e.g. IL4) in the same paper. Closely related to GN is the important and fundamental task of identifying proteins for inclusion in protein-protein interaction (PPI) databases. PPIs are of great interest to biomedical researchers because of their crucial role in elucidating signal pathways, controlling central biological processes such as transcription factors involved in cell division and DNA transcription [1], and their implications in a range of human diseases including cancer and neurodegeneration [2,3]. To provide efficient widespread access to PPIs information, some organizations have begun compiling structured PPI annotation in public databases. MINT [4], IntAct [5], and BioGRID [6] are examples of PPI databases containing large numbers of verified PPIs. PPI databases are also very useful in building databases of signalling pathways, like BioCarta [3], or protein networks.

Since most PPI information exists in published papers, text mining is an ideal way to speed up construction of these databases. Several research institutes and groups have been active in the effort to curate resources for PPI text mining and lay our roadmaps for the development of PPI mining tools. In 2005, BioCreAtIvE I/II [7,8], held by the Centro Nacional de Investigaciones Oncológicas (CNIO), established the GN task, in which participant systems must map gene names to database identifiers. Then in 2009, CNIO launched the interactor normalization task (INT) [9,10] at the BioCreAtIvE II.5 challenge. Another more important and difficult task in the BioCreAtIvE II.5 challenge is the interaction pair task (IPT). In the IPT task, systems are further asked to return a list of interaction identifier pairs for each article.

The goal of IPT is to map interaction pairs in well-formed full-text articles to UniProt identifiers (see [9,10] for details on IPT) and to rank these identifier pairs according to their probability of being interactors (refers to the Interactor Ranking for detail). Ranking PPI pairs is very important because only a small percentage of the identifiers are suitable for database curation (23.10% in abstracts, and 7.02% in full-text articles). In a quality ranked list, the curatable identifier pairs should be placed at the top. Such a ranked list would be more useful for human curators.

**Figure 1:**
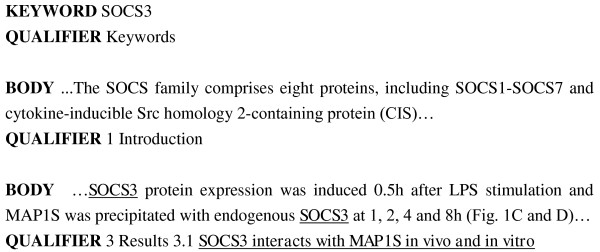
**Snippet of a full text article in the BioCreAtIvE II.5 dataset**.

Unlike other biomedical text mining tasks, which use only abstracts as research data, the BioCreAtIvE II.5 challenge compiled a dataset of full-length journal articles, which were formatted in a well-formed block structure. Figure 1 shows an example article with three blocks. The first line of each block defines the section type and its corresponding text content. The section types include TITLE, ABSTRACT, and BODY for the main article content, as well as FIGURE, KEYWORD, and TABLE. The second line defines the section and the subsection headings. For example, the article has a keyword "SOCS3", and a subsection heading "SOCS3 interacts with MAP1 S in vivo and in vitro".

Our three main contributions in this paper are a section-optimized ranking model, an efficient dynamic-programming-based relational re-ranking algorithm, and the interaction pair ranking function. Firstly, our ranking model is optimized to take advantage of section-specific information. Several studies [11-13] have shown that scientific authors do, in the majority of cases, follow the basic principles of the research article structure and assign information accurately to each section. Each section of the paper has different characteristics which we can use to guide GN and the ranking algorithm. For example, the Introduction often contains information that repeatedly appears throughout the article (key genes), while the Results section presents new scientific findings, such as PPIs. In addition to section-specific information, our ranking model also makes use of the metadata that is included in full-text articles, such as the keywords fields.

Secondly, we have developed a re-ranking algorithm that considers co-occurrence among interactors in an article. Co-mentioned genes influence each other's rank [14,15]. If two gene names frequently occur alongside each other in the same sentences in an article, they are likely to have an influence on each other's rank. Take a low-ranked interactor mentioned only twice in an article for example. If both mentions happen to be alongside the highest-ranked interactor in the article, then the low-ranked interactor's rank should be significantly boosted. Our re-ranking algorithm is designed to use this information to improve accuracy of interactor ranking. Using a greedy computational approach, the re-ranking procedure requires large amounts of computer resources and time to calculate each gene identifier's rank simultaneously and find the best ranked list. Therefore, to maximize computational efficiency, we implemented our re-ranking algorithm using dynamic programming.

Finally, we describe an approach to generating a ranked list of binary interaction pairs which combines the INT re-ranking results with a labor-saving unsupervised approach that still achieves competitive results.

## Results and discussion

### Implementation

#### Overview of the proposed INT and IPT system

**Figure 2:**
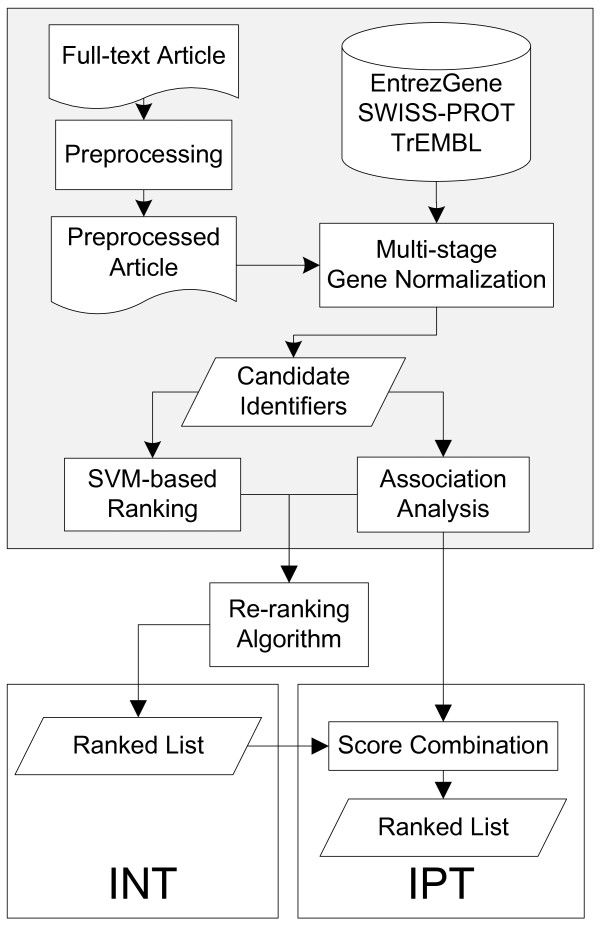
**System workflow**.

Figure 2 shows a flowchart of our system for INT and IPT. The top block depicts the fundamental steps for both tasks. The well-formed full-text article is preprocessed to resolve the conjunction problems presented by [16]. We use several rules proposed in our previous work [17] to expand collapsed ranges, such as SOCS1-SOCS7 in the Introduction section of Figure 1, into their individual components.

After preprocessing, the multi-stage GN procedure [18] is executed (see "Multi-stage gene normalization" sub-section). For each identifier extracted by the multi-stage processing, the corresponding context information is used to extract features and the identifier is ranked by a support vector machine (SVM) classifier (see "SVM-based ranking model" sub-section).

In order to further refine the ranking results, we create a re-ranking algorithm that takes into consideration the rank of an identifier and the genes that co-occur alongside. The re-ranking results are treated as an interaction candidate list for the article.

To further extract interaction pairs, an unsupervised association analysis method is employed to determine their association scores. We then combine the scores with the results of INT re-ranking to generate the list of interaction pairs (see "Using re-ranked INT results for IPT" section).

### Algorithm

#### SVM-based ranking model

Our model is based on the SVM algorithm, a well-known Machine Learning (ML) algorithm that has proved useful for text classification [19]. We have extracted the following features related to rank identifiers found in the GN procedure:

##### Frequency features

The frequency with which the identifier appears in the entire article is used as a feature. In addition, based on the work of [20], who found that molecular interaction descriptions usually appear in the Results section, we added the percentage of an identifier found in the Results section against in the other sections as a feature.

##### Location features

The features indicate where the identifier appears in the full text. The following location types are taken into consideration: (1) title; (2) abstract; (3) the first section (usually the Introduction section), the Results section, the last section (usually the Conclusion section), the Appendix section, and the other sections; (4) section and sub-section titles, such as "3 Results 3.1 SOCS3 interacts with MAP1 S in vivo and in vitro" in the Figure 1; and (5) Figure and Table captions.

##### Field features

These features indicate if the target identifier's name occurs in the full-text article's Keyword field, full name, or abbreviation field.

##### Co-occurrence features

These features describe co-occurrence of the target interactor with three types of the paper's key information: keywords, full names/abbreviations, and references to figures in the article text. Co-occurring with the paper's keywords or full names/abbreviations implies that the identifier is very likely to be key identifier in this article [21] and [22] have shown that figures often concisely summarize the most important results or methods used and described in an article. The paragraphs containing figure references often summarize the content of a figure. Therefore it follows that identifiers that co-occur beside figure references have a higher possibility of being interactors. For instance, the interactor "SOCS3" appears beside "Figure 1C and 1D" in the Figure 1.

##### Indicator phrase features

[23] and [24] have shown that there are commonly occurring structures which explicitly state the article's main knowledge claim or the assertion for which the authors hope to be cited and credited in future articles. Examples are "the aim/purpose of this paper/article/study" and "we conclude/propose". Those phrases that signal important sentences in a text are referred to as indicator phrases [25,26]. Such sentences can be used to create an extract-type summary of the text. We add a feature to indicate whether or not the identifier occurs in the same sentences that contain indicator phrases.

Table 1 shows an example of the features used for SVM-based ranking and a sample ranking result, *r *= [P40337, P40338, Q05513, Q9NPB6].

**Table 1:** Re-ranking example

Gene ID	P40337	P40338	Q05513	Q9NPB6
GN procedure	Exact Match	Exact Match	Exact Match	Exact Match
frequency	45/0.76	14/0.00	10/0.20	7/0.57
location	Title, first, Results, last, sub-section title, fig	Abstract, fig	Abstract, first, Results, last	first, Results, last
co-occurrence	keywords, abbr-full, fig	keywords, abbr-full	keywords	
indicator phrase	✓	✓		✓

SVM ranking result	1	2	3	4

Concurrence information	P40338:1 Q05513:2 Q9NPB6:2	P40337:1	P40337:2 Q9NPB6:1	P40337:2 Q05513:1

#### Re-ranking algorithm based on co-mentioned genes

The ranking procedure tends to rank the focus genes higher. However, the main concern of INT and IPT is to extract interactors. Our re-ranking algorithm is designed to boost the rank of interactors by considering co-occurrence among gene mentions in a single article. The example shown in Table 1 gives a general illustration of how the algorithm works.

Table 1 shows the initial ranking results, *r *= [P40337, P40338, Q05513, Q9NPB6], determined by SVM. The algorithm starts by using a function, **newRankedList **(see "Step1: Generate possible ranked lists" in Methods). Given the rank-*i *identifier *x *and all other identifiers *y *in *r *that co-occur with *x*, **newRankedList **generates a ranked list *l_x_*, where the 1^st ^to *i*-1^th ^position are empty slots, *x *is put in the *i*th slot. Each *y *is put in a slot starting at *i*+1^th ^from highest to lowest **association**(*x*,*y*) score. The remaining slots are empty slots. The generated lists for Table 1 are shown in Table 2. We use an unsupervised approach based on a sentence-level mutual information (MI) [27] to measure the association (see Methods section) because the dataset provided by the BioCreAtIvE II.5 challenge did not contain sentence-level PPI pair annotation. In this example, the new ranked list generated by P40337 is [P40337, Q9NPB6, Q05513, P40338]. For P40338, the ranked list is [_, P40338, P40337, _]. The others are excluded because P40338 only co-occurs with P40337.

**Table 2:** Possible ranked lists determined by all identifiers

Gene ID\Rank	1	2	3	4
P40337	P40337	Q9NPB6	Q05513	P40338
P40338		P40338	P40337	
Q05513			Q05113	Q9NPB6
Q9NPB6				Q9NPB6

After generating all possible ranked lists, there are new candidates in each rank. As shown in Table 2, Q9NPB6 and P40338 are candidates in rank 2 and 4 respectively. Therefore, there are several possible combinations and each one represents a possible re-ranked list. We then define a **score **function that estimates the likelihood of an identifier *x *being re-ranked in *i *as follows:

$$\begin{array}{l} \text{score}(x,i,\text{L})=\\ \text{rankN}\_\text{Ratio}(x,i,\text{L})\times \text{svmAccuracy}(i)\times \\ \text{svmAccuracy}(\text{deciderRank}(x,i,\text{L})) \end{array}$$

where the **rankN_Ratio **function is calculated based on all possible ranked lists, **L **(see "Step 2: Assign scores to identifiers in all possible ranked lists" for the details); **svmAccuracy **is the function that returns the INT accuracy of rank *i *in our SVM ranking (Details and rank accuracies are listed in "Preliminary experiments on the INT training set" section); the **deciderRank **function returns the rank of the highest ranked identifier that supports an identifier *x *in rank *i*. For example, assuming that the identifier *w*'s corresponding new list is [*w*, *x*, *y*, *z*] and *x*'s is [_, *x*, *y*, *w*], *y *will be ranked third, and **L **= {[*w*, *x*, *y*, *z*], [_, *x*, *y*, *w*]}. In this case, **deciderRank(***y*,3,**L**), will return 1 since *w *and *x *support *y *in rank 3, but *w*'s rank is higher than *x*'s.

For a possible re-ranked list *r*, its overall score can be calculated as follow:

$$\text{overallscore}(r',\text{L})=\prod_{i=1}^{\left|r'\right|}\text{score}(r\text{'[}i\text{],}i\text{,}\text{L})$$

where *r*'[*i*] is the *i*th element of *r*'. The re-ranked list with the highest **overallscore **among all possible combinations is chosen as the final output re-ranked list.

If the duplication of identifiers in a re-ranked list is permitted, the optimal ranked list can be directly found by choosing the identifier with the highest ***score ***value for each rank. However, a legal ranked list cannot have any duplicates. To avoid duplication, we add a duplication constraint on the **score **function: when estimating **score**(*x*,*i*,**L**), if the identifier *x *has been determined in the previous rank, *k*, the **score**(*x*, *k*,**L**) function must return 0 (i.e. **overallscore**(*x*,*k*,**L**) is 0). Unfortunately, the duplication constraint increases the computational complexity of finding the optimal ranked list. In order to find the optimal ranked list and avoid computational overhead, we propose a dynamic-programming-based algorithm. Details on the algorithm are described in the "Implementation of the re-ranking algorithm" sub-section.

#### Using re-ranked INT results for IPT

In order to extract interaction pairs, we follow the workflow shown in Figure 2 to process the article and generate a ranked list of identifiers. If two identifiers are described in one sentence, they are extracted as an interaction pair candidate. The mutual information (MI) analysis described in the Methods section is used to determine their association score. We then combine the association analysis with the results of INT ranking. Based on the assumption that the **IPTScore **(which estimates if *x *and *y *is an interactor pair given *x *in rank-*i *and *y *in rank-*j*) is positively relevant to **association**(*x*,*y*) and *x *and *y*'s individual interactor scores, we propose the following formula to combine the results of association analysis and re-ranking:

IPTScore(x,i,y,j)=association(x,y)×interactor_score(x,i)×interactor_score(y,j)

When using the **score **function introduced in Equation 1 as the **interactor_score **function, the formula can be rewritten as:

IPTScore(x,i,y,j)=association(x,y)×score(x,i,L)×score(y,j,L)

Because we use MI to determine the association, the formula can be rewrite as follows:

IPTScore(x,i,y,j)=MI(x,y)×score(x,i,L)×score(y,j,L)

### Testing

#### Dataset and evaluation metrics

The BioCreAtIvE II.5 [28] provides 124 journal articles selected mainly from FEBS Letters for evaluating INT and IPT systems. Following the format of the BioCreAtIvE II.5 challenge, we use 61 articles published in 2008 (50%) as our training set and 63 articles published in 2007 or earlier (50%) as our test set. The dataset is preprocessed to convert Greek alphabet characters (e.g. α, β) to corresponding diacritics (e.g. alpha, beta).

The most common information extraction (IE) evaluation metric is centered on F-measure, an evaluation score generated from combining precision and recall. This evaluation has an obvious shortcoming: it does not take ranking of results into account. E.g., two systems reporting the same two correct and eight wrong hits for a document would produce the same F-scores, including precision and recall values, no matter the ranking of the results. Therefore, the area under curve (AUC) [29] of the interpolated precision/recall (iP/R) curve used in the BioCreAtIvE II.5 challenge is used to evaluate the proposed approach. The AUC of the iP/R function *f_pr _*is defined as follows:

AUC iP/R(fpr)=∑j=1n(pij×(ri–ri–1))pi(r)=maxr'≥rp(r)

where *n *is the total number of correct hits and *p_i _*is the highest interpolated precision for the correct hit *j *at *r_j_*, the recall at that hit. Interpolated precision *p_i _*is calculated for each recall *r *by taking the highest precision at *r *or any *r*' ≥ *r*.

#### Preliminary experiments on the training set

To examine calculate the accuracies used in the **score **function (Equation 1), we apply the thirty-fold cross validation on the training set. We use the SVM-based ranking procedure to determine ranks, and then calculate each rank's accuracy using the training set. The accuracy of rank *i *is calculated by the following formula:

$$\text{svmAccuracy}(i)=\frac{\text{the number of correctly nomalized identifiers in rank }r}{\text{the number of normalized identifiers in rank }r}$$

**Figure 3:**
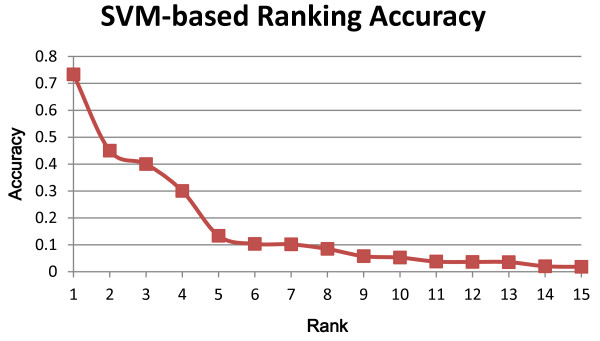
**SVM-based ranking accuracy across different ranks in the training set**.

Figure 3 shows the accuracies of ranks 1 to 15. We can see that as the rank increases, the accuracy drops. This implies that higher ranks predicted by our SVM-based ranking method are more reliable than lower ranks. As mentioned in Equation 1, we utilize this phenomenon for scoring each identifier. After all ranks' accuracies are calculated, the re-ranking algorithm is employed.

#### INT test set performance

**Figure 4:**
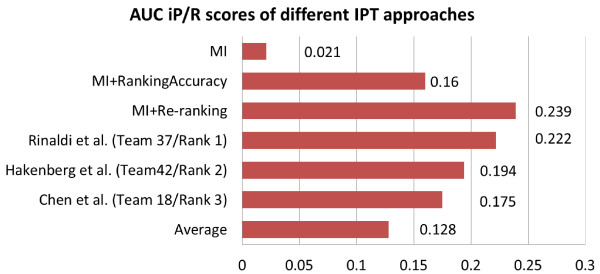
**AUC iP/R scores of different INT approaches**.

Figure 4 shows the AUC iP/R scores of three configurations of our system. In the configuration (SVM ranking/Rank1), multi-stage GN and SVM-based ranking are employed. In the configuration (SVM ranking+Re-ranking), the proposed re-ranking algorithm is added. We also implement a baseline method (Freq) which ranks all identifiers according to their frequency. If two or more identifiers have the same frequency, two criteria are employed sequentially to rank them: (1) highest frequently in the Results sections (2) mentioned earliest in the article. Lastly, Figure 4 also shows the AUC iP/R scores of the top three teams and the average AUC iP/R score of all BioCreAtIvE II.5 INT participants (Average).

As shown in Figure 4, after employing our re-ranking algorithm, AUC iP/R performance increases by 1.84% over the previous top score in BioCreAtIvE II.5. According to our analysis, before re-ranking, gene identifiers whose feature values rarely appear in the training set are often incorrectly ranked because their feature values are underweighted in the ranking model. However, if these identifiers co-occur with higher-ranked identifiers whose feature values frequently appeared, our re-ranking algorithm is very likely to increase their ranks. This results in the improved AUC iP/R score.

#### IPT test set performance

**Figure 5:**
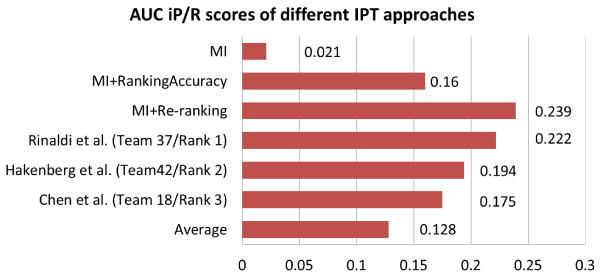
**AUC iP/R scores of different IPT approaches**.

Figure 5 compares the results of three configurations of our IPT system. In the first configuration (MI), we rank all possible interaction pairs according to their MI scores. In the second configurations (MI+svmAccuracy), we simplify the original Equation 2 to rank all pairs. The **score **function is replaced by **svmAccuracy**. In the third configuration (MI+Re-rank), we use Equation 2 to rank all pairs. Figure 5 also shows the AUC iP/R scores of the top three BioCreAtIvE II.5 IPT teams and the average AUC iP/R score of all BioCreAtIvE II.5 IPT teams (Average).

As shown in Figure 5, MI achieves a very low AUC iP/R (2.07%), while MI+SVMAccuracy achieves a much better AUC iP/R (16.02%) than Average (12.80%). MI+Re-ranking (our proposed method) further achieves a competitive AUC iP/R of 23.86% (outperforms the rank-1 system in BioCreAtIvE II.5 IPT). The results show that by using the results of INT re-ranking, the AUC iP/R score can be improved by 7.84% (compared to our system without re-ranking MI+SVMAccuracy) Comparing with the top team [10,30,31], which constructed a syntactic filter by semi-automatically annotating a PPI syntactic path corpus based on the GENIA corpus [32], and the third team [33], which manually annotated interaction proteins according to SDA information to construct a corpus for supervised learning, our approach requires less annotated data to construct and has the potential to be improved by using advanced **association**(*x*,*y*) calculation techniques.

#### Comparison of SVM-based ranking and re-ranking

We apply a two-sample *t*-test to examine whether the proposed re-ranking method is better than the original SVM-based ranking method by a statistically significant difference in INT and IPT. The null hypothesis, which states that there is no difference between the two configurations A and B, is given as

$$H_{0}:\mu_{A}=\mu_{B}.$$

where *μ_A _*is the true mean AUC iP/R of configuration A, *μ_B _*is the mean of the configuration B, and the alternative hypothesis is

$$H_{1}:\mu_{A}>\mu_{B}.$$

A two-sample *t*-test is applied since we assume the samples are independent. As the number of samples is large and the samples' standard deviations are known, the following two-sample *t*-test is suitable:

$$t=\frac{\left({\bar{X}}_{A}-{\bar{X}}_{B}\right)}{\sqrt{\frac{s_{A}^{2}}{n_{A}}+\frac{s_{B}^{2}}{n_{B}}}}$$

If the resulting *t*-score is equal to or less than 1.67 with a degree of freedom of 89 and a statistical significance level of 95%, the null hypothesis is accepted; otherwise it is rejected.

To get the average AUC scores and their deviations required for the *t*-test, we randomly sampled ninety training sets (*g*_1_,..., *g*_90_) and ninety test sets (*d*_1_,..., *d*_90_) from the 124 BioCreAtIvE II.5 journal articles. We trained the model with baseline ranking on *g*_i _and tested it on *d*_i_. We then performed the re-ranking procedure on the test results for *d*_i_. Following that, we summed the scores for all ninety test sets, and calculated the averages for performance comparison. Table 3 shows the results. We can see that after re-ranking, INT and IPT performances are improved by 3.12% and 3.88% respectively on the AUC iP/R scores with a statistically significant difference.

**Table 3:** Comparison of INT and IPT performance on SVM-based ranking and SVM-based ranking + re-ranking

	Avg AUC iP/R(%)		Stdev AUC iP/R(%)		*t*	*t *> 1.67?
		
	SVM	SVM+ Re-ranking	SVM	SVM+ Re-ranking		
INT	47.29	50.41	6.30	6.34	3.31	Y

IPT	15.87	19.75	6.39	6.85	3.93	Y

## Conclusions

In this paper, we have proposed a SVM-based ranking procedure with a relational re-ranking algorithm that considers the associations among gene identifiers to further improve performance on the BioCreAtIvE II.5 INT and IPT task. We formulated the re-ranking problem as an optimization problem and solved it by using dynamic programming to reduce computational complexity.

We evaluated our approach on the BioCreAtIvE II.5 full-text dataset. In INT, the highest AUC iP/R achieved by our re-ranking system is 45.34%, 1.84% higher than that of our SVM-based system (Rank 1 in the BioCreAtIvE II.5 INT challenge). In IPT, our unsupervised method incorporating re-ranking not only achieves a promising AUC iP/R of 23.86%, which exceeds the best score in the BioCreAtIvE II.5 IPT challenge by 1.64%, but also saves significant annotation effort in comparison to other top teams' supervised methods. A statistical significance *t*-test using ninety randomly selected training/test sets confirms that our additional re-ranking procedure significantly improves performance over the baseline ranking method.

The proposed re-ranking algorithm relies heavily on association information, which it uses to generate possible ranked lists as described in the Algorithm section. We believe that our proposed approach could be combined with other advanced association scoring methods to further improve results. In the future, we plan to integrate our relation extraction method, which was developed for extracting hypertension-related genes in [34], into our INT and IPT system, and study the performance gains. We will also continue to improve our online web service to allow users to upload full text articles for PPI pair extraction.

## Methods

### Multi-stage gene normalization

We have updated our previous one-stage GN system [17] with keyword-based species determination processing as well as multi-stage processing. In the following paragraphs, we briefly describe the improvement. Please refer to [18] for details.

Keyword-based species determination finds the species for a given gene by checking the surrounding text for species keywords. If keywords are found, the corresponding identifier is assigned, otherwise the most frequently described species in the article is chosen.

In order to exploit the characteristic of difference sections, we propose a three-stage GN procedure. In the first stage, GN is carried out on the Title, Abstract and Introduction sections. Successfully normalized gene mentions and corresponding identifiers are collected into a dictionary. In the second stage, we search the whole article for mentions recorded in this dictionary. The Title, Abstract, and Introduction sections are rechecked in case our machine learning (ML)-based gene mention tagger [35,36] missed any instances. In the third stage, the remaining paper sections (except Title, Abstract, and Introduction) including figure/table captions and appendix descriptions are processed by our GN system. When combined with the dictionary-based approach used in stage two, disagreement of boundaries or identifiers may occur. In such cases, we select the candidate identifier with the longest gene mention string.

#### Implementation of the re-ranking algorithm

**Figure 6:**
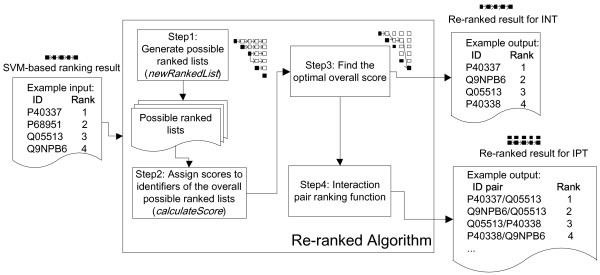
**The re-ranking algorithm**.

Figure 6 shows the main steps of the proposed re-ranking algorithm. It accepts the output of the ranking procedure mentioned in "SVM-based ranking model" section as its input, and generates the re-ranked list as its output. In the following paragraphs, we describe the algorithm in detail.

### Notation

Let *r *be the ranked list generated by the SVM ranking procedure and *x *be any identifier in *r*. The possible ranked list generated by *x *is denoted by *l_x_*. The set of all possible ranked lists is denoted as **L **= {*l_x_*|*x *in *r*}. For a possible re-ranked list, *r*', the rank-*i *identifier is denoted as *r*'[*i*].

#### Step 1: Generate possible ranked lists

**Figure 7:**
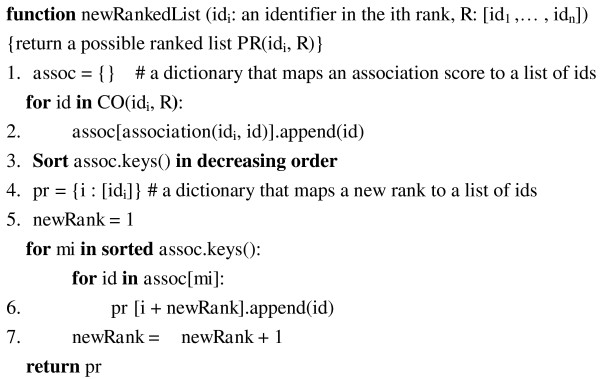
**A pseudo code implementation for *newRankedList *function**.

In the following paragraphs, we describe the algorithm, **newRankedList**, and functions that are used for generating possible ranked lists. A pseudo code implementation of the algorithm using python syntax is shown in Figure 7.

**association**(*x*,*y*):

This function measures the association between an identifier (interactor) *x *and another identifier *y *within an article and returns an association score. Several approaches can be used to measure this score, such as rule learning [37,38], co-citation analysis [39], maximum entropy model [40], and conditional random fields [41]. Because the dataset provided by the BioCreAtIvE II.5 challenge did not contain sentence-level PPI pair annotation, we use an unsupervised approach based on mutual information (MI) [27] to measure the association:

MI(x,y)=P(x,y)/(P(x)×P(y))

In the above formula, *P*(*x*) and *P*(*y*) are estimated by dividing *x*'s and *y*'s frequencies by *N*, the number of sentences containing gene identifiers. The joint probability, *P*(*x*,*y*), is estimated by dividing the frequency that *x *co-occurs with *y *in the same sentence by *N*.

**newRankedList**(*x*,*i*)**:**

Give the rank-*i *identifier *x *and all other identifiers *y *in *r *that co-occurs with *x*, **newRankedList **generates a ranked list *l_x_*, where the 1^st ^to *i*-1^th ^position are empty slots, *x *is put in the *i*th slot. Each *y *is put in a slot starting at *i*+1^th ^from highest to lowest **association**(*x*,*y*) score (see Figure 7, lines 5-7). The remaining are empty slots. Take a ranked list of four identifiers *w*, *x*, *y *and *z *for example. For the rank 2 identifier *x*, the output of **newRankedList**(*x*,2) will be [_, *x*, *z*, *y*] if **association**(*x*,*z*) >**association**(*x*,*y*).

#### Step 2: Assign scores to identifiers in all possible ranked lists

Given an identifier *x*, a rank *i *and the set of possible ranked lists **L **generated in step 1, the score of *x *in *i*th rank is calculated as follows:

score(x,i,L)=rankN_Ratio(x,i,L)×svmAccuracy(r)×svmAccuracy(deciderRank(x,r,L))

The following are definitions of functions used for defining **score**:

**svmAccuracy**(*i*):

Given a rank *i*, the function returns the INT accuracy of rank *i *in our SVM ranking. The accuracy is calculated based on a three-fold cross validation carried out on the training set.

**deciderRabk**(*x*,*i*,**L**):

For an identifier *x *whose new rank *i *is determined by more than one identifier, the function returns the rank of the highest ranked identifier. If only one identifier, *y*, determines *x *in *i*, the function returns the rank of *y*.

**rankN_Ratio**(*x*,*i*,**L**):

Given an identifier *x*, rank *i *and the set of all possible ranked lists **L**, the **rankN_Ratio **score is calculated based on the following equation:

$$\text{rankN}\_\text{Ratio}(x,i,\text{L})=\frac{\sum_{y}\text{isRankN}(x,i,l_{y})}{\sum_{y}\sum_{j=1}^{\left|r\right|}\text{isRankN}(x,j,l_{y})}$$

where *y *is any identifier except *x *in *r*

The intuition behind **rankN_Ratio **is that the more ranked lists that agree with *x *in rank *i*, the more likely it is that *x *is in rank *i*.

**isRankN**(*x*,*i*,*l_y_*) indicates whether the identifier *x *is in rank *i *of *l_y _*(return 1) or not (returns 0). Therefore, $\sum_{y}\sum_{j=1}^{\left|r\right|}\text{isRankN}(x,j,l_{y})$ is the total number of times *x *appeared in all ranks of all lists in **L**.

**Figure 8:**
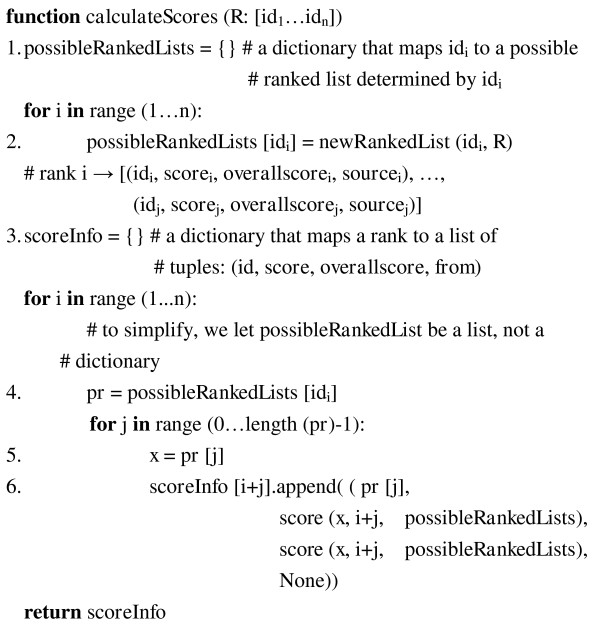
**Pseudo code implementation of *calculateScores *function**.

Figure 8 shows the algorithm illustrated in step 2. It first generates all possible ranked lists for each identifier in *r *(lines 1-2). For each rank, the corresponding identifiers and their scores are calculated and stored in a dictionary-like data structure, *scoreInfo *(lines 3-6). Table 4 shows the data structure and its attributes.

**Table 4:** The *scoreInfo *data structure

*scoreInfo*
A dictionary maps the rank (an integer) to a list of tuples: (*id*, *score*, *overallscore*, *from*). The dictionary's keys are the ranks in the re-ranked list.
**Attributes**
tuple.id: the identifier
tuple.score: the score of tuple.id
tuple.overallscore: the overall score of the ranked list after considering tuple.id
tuple.from: the identifier in the previous rank, which leads to the optimal
tuple.overallscore
**Methods**
*scoreInfo*[*key*]: Return the list of tuples in *si *with key *key*.
*scoreInfo *[*key*][*i*]: Return the *i*th tuple in the list in *si *with key *key*.

#### Step 3: Find the optimal overall score

Given a possible re-ranked list, *r*', its score is defined as follows:

$$\text{overallscore}(r',\text{L})=\prod_{i=1}^{\left|r'\right|}\text{score}(r'[i],i,\text{L})$$

where *r*'[*i*] is the *i*th element of *r'*.

We can now formulate the re-ranking problem as an optimization problem that maximizes the overall scores over all possible rank orders:

argmaxr'  overallscore(r',L)

The duplication constraint on the **score **function can be defined as follows: when estimating **score **(*x*,*i*,**L**), if the identifier *x *has been determined in the previous rank, *k*, the **score **(*x*,*k*,**L**) function must return 0 (i.e. **overallscore**(*x*,*k*,**L**) equals 0). For example, consider two possible re-ranked lists: *r*_1 _= [*x*, *w*, *y*,...] and *r*_2 _= [*x*, *y*, *z*,...]. Assuming that **overallscore**(*r*_1_,**L)> overallscore**(*r*_2_,**L)**, and we now want to determine the value of **overallscore **function when *w *is in rank 4, then *r*_1_'s **overallscore **becomes 0 because **score**(*w*,2,**L**) = 0 and *r*_2_'s **overallscore **is greater than zero. Therefore, even though in rank 3, *r*_1_'s **overallscore **is higher than *r*_2_'s, the algorithm will not choose *r*_1 _as the optimal sub-ranking when considering *w *in rank 4.

**Figure 9:**
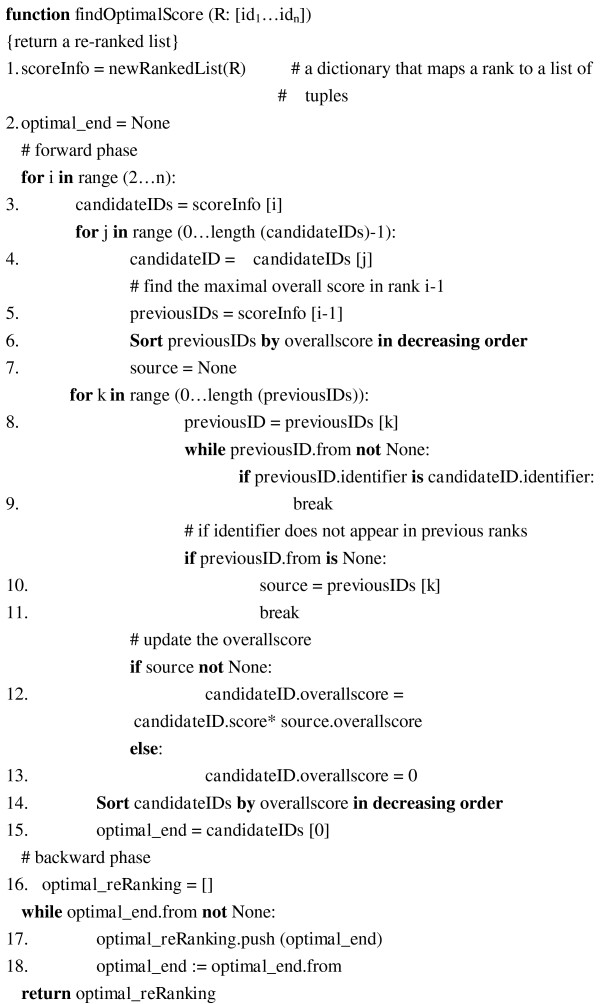
**Pseudo code implementation for *findOptimalScore *function**.

Figure 9 shows the dynamic-programming-based re-ranking algorithm. The algorithm starts by using *calculateScores *defined in Step2 to generate all possible ranked lists and their scores, which are stored in the data structure *scoreInfo *(lines 1). Lines 2-18 of Figure 9 encompass the dynamic programming approach employed to find the optimal ranked list.

In the forward phase (lines 3-15), the algorithm computes the optimal overall score for each identifier in each rank. Lines 8-11 find the maximum overall score in *scoreInfo*[*i*-1] in which the identifier, *scoreInfo*[*i*][*j*] = *candidatesIDs*[*j*], does not appear among rank 1 to rank *i*-1.

In the following formula, *scoreInfo*[*i*][*j*].overallscore is shorted to **overallscore **(*i*,*j *), which is the optimal overall score from rank 1 to rank *i *when rank *i*'s *j*th candidate is placed at rank *i*. *scoreInfo*[i][j].identifier is shorted to, which stands for rank *i*'s *j*th candidate. The score can be recursively calculated as follows:

−{score(ID(i,j),i,L)×max{overallscore(i−1,0)⋮overallscore(i−1,k−1)} if i>2score(ID(1,0),1,L) if i=1,j=0

where *k *is the number of tuples in the *scoreInfo*[*i*-1]. Lines 12-13 calculate the score.

In the backward phase (lines 16-18), the optimal ranking is reconstructed by tracing the "from" attribute of the tuple in the last rank with maximal overall score (the *optimal_end*) until the value of "from" is None.

#### Step 4: Interaction pair ranking function

After Step3, the algorithm has generated the re-ranked list for INT. The final step of the algorithm is to employ the IPTScore function defined in Equation 2 to generate the ranked list for IPT.

## Availability and requirement

We have developed a demo website to demonstrate the proposed re-ranking algorithm. The service is available at http://biosmile.cse.yzu.edu.tw/DPRerankAlgorithmForAbstractDemoWebsite/. The web-based service has been tested and run on the Firefox 3.5+, Chrome 7+, and IE7+.

## Authors' contributions

RTHT designed all the experiments and wrote most of this paper. PTL wrote the programs and conducted all experiments. RTHT guided the whole project. All authors approved the manuscript.
